# Genome-wide association study for metabolic syndrome reveals *APOA5* single nucleotide polymorphisms with multilayered effects in Koreans

**DOI:** 10.1186/s12944-024-02248-0

**Published:** 2024-08-28

**Authors:** Young Jun Park, Sungji Moon, Jaeyong Choi, Juhyun Kim, Hyun-Jin Kim, Ho-Young Son, Sun-Wha Im, Jong-Il Kim

**Affiliations:** 1 Genomic Medicine Institute, Medical Research Center, Seoul National University, Seoul, 03080 Republic of Korea; 2 Department of Translational Medicine, Seoul National University College of Medicine, Seoul, 03080 Republic of Korea; 3 Interdisciplinary Program in Cancer Biology, Seoul National University College of Medicine, Seoul, 03080 Republic of Korea; 4 Cancer Research Institute, Seoul National University, Seoul, 03080 Republic of Korea; 5 Department of Biomedical Sciences, Seoul National University College of Medicine, Seoul, 03080 Republic of Korea; 6 National Cancer Control Institute, National Cancer Center, Goyang-Si, Gyeonggi-Do 10408 Republic of Korea; 7 Department of Biochemistry and Molecular Biology, Seoul National University College of Medicine, 103 Daehak-ro, Jongno-gu, Seoul, 03080 Republic of Korea; 8 Department of Biochemistry and Molecular Biology, Kangwon National University School of Medicine, One Kangwondaehak-gil, Chuncheon-si, Gangwon-do, 24341 Republic of Korea

**Keywords:** Metabolic syndrome, Apolipoprotein A5, Genome-wide association study, Korean genome and epidemiology study, Triglycerides

## Abstract

**Background and purpose:**

Genome-wide association studies (GWAS) of metabolic syndrome (MetS) have predominantly focused on non-Asian populations, with limited representation from East Asian cohorts. Moreover, previous GWAS analyses have primarily emphasized the significance of top single nucleotide polymorphisms (SNPs), poorly explaining other SNP signals in linkage disequilibrium. This study aimed to reveal the interaction between rs651821 and rs2266788, the principal variants of apolipoprotein A5 (*APOA5*), within the most significant loci identified through GWAS on MetS.

**Methods:**

GWAS on MetS and its components was conducted using the data from the Korean Genome and Epidemiology Study (KoGES) city cohort comprising 58,600 individuals with available biochemical, demographic, lifestyle factors, and the most significant *APOA5* locus was analyzed further in depth.

**Results:**

According to GWAS of MetS and its diagnostic components, a significant association between the *APOA5* SNPs rs651821/rs2266788 and MetS/triglycerides/high-density lipoprotein phenotypes was revealed. However, a conditional analysis employing rs651821 unveiled a reversal in the odds ratio for rs2266788. Therefore, rs651821 and rs2266788 emerged as independent and opposing signals in the extended GWAS analysis, i.e., the multilayered effects. Further gene-environment interaction analyses regarding lifestyle factors such as smoking, alcohol consumption, and physical activity underscored these multilayered effects.

**Conclusion:**

This study unveils the intricate interplay between rs651821 and rs2266788 derived from MetS GWAS. Removing the influence of lead SNP reveals an independent protective signal associated with rs2266788, suggesting a multilayered effect between these SNPs. These findings underline the need for novel perspectives in future MetS GWAS.

**Supplementary Information:**

The online version contains supplementary material available at 10.1186/s12944-024-02248-0.

## Introduction

The National Cholesterol Education Program Adult Treatment Panel III (NCEP-ATP III) guideline defines metabolic syndrome (MetS) as an amalgam of metabolic abnormalities, including abdominal obesity, increased blood sugar levels, elevated blood pressure, and dyslipidemia [1]. Based on this definition, the prevalence of MetS was 34.2% in the United States and has been reported to increase throughout the 21st century [2]. In a nationwide Korean survey, approximately 30% of the population was diagnosed with MetS [3]. MetS is clinically important because it is associated with a 2-fold increased risk for cardiovascular diseases such as coronary artery disease and stroke as well as a 5-fold increased risk of type 2 diabetes [4]. As the prevalence of obesity and MetS has increased over the years, the healthcare costs for targeting these diseases have increased. Numerous potential treatments for MetS have been proposed; however, their effectiveness has been relatively moderate, thereby emphasizing the need for advanced precision medicine for clinical applications [5].

Family-based studies have estimated the heritability (*h*^*2*^) for each component of MetS to be 30–50% [6], with the remaining risk attributed to lifestyle and environmental factors such as diet, physical activity, and other non-genetic influences. The largest genome-wide association study (GWAS) on MetS has identified lipid-related (*ZPR1*,* CETP*, and *LPL*), obesity-related (*FTO*,* MC4R*, and *VEGFA*), diabetes-related (*SLC18A1*,* ARAP2*, and *EXOC6*), and hypertension-related (*SIPA1*) genes, and genes with pleiotropic effects simultaneously affecting two or more MetS components (*GALNT2*,* FADS2*,* NEU2*, and *CMIP*) [7].

Although previous GWAS have provided valuable insights into the genetic background of MetS, the unexplained heritability remains to be explored. One hypothesis that could explain this is that the contribution of individual variants to the phenotype has not been fully elucidated, possibly owing to limitations in statistical analyses considering linkage disequilibrium (LD). During the interpretation of GWAS results, only a small number of significant single nucleotide polymorphisms (SNPs) that are not within an LD relationship with each other have been selected for each locus, whereas the remaining SNPs have been ignored. However, certain variants that are located nearby within a locus, with a delicate LD relationship with each other, could exhibit independent effects on the phenotype [8]. Moreover, massive parallel reporter assays have revealed that genetic association signals can present through multiple tightly-linked causal variants [9].

Most of the previous GWASs on MetS have been conducted in non-Asian populations, and relatively few involved Korean populations [7, 10, 11]. The heterogeneous characteristics of MetS across populations make it challenging to identify the SNPs that contribute to the phenotype, let alone discover novel SNPs [12]. Certain ethnic groups, such as Hispanics or African Americans, may have a relatively high prevalence of MetS [13]. Furthermore, the prevalence and severity of specific MetS components may differ among populations. For instance, insulin resistance may be more prevalent in South Asians [14], whereas high blood pressure may be more pronounced in African Americans [15].

Therefore, this study strived to perform a GWAS on MetS and its components in a large Korean cohort to identify numerous significant loci that influence metabolic factors, and further investigate the role of SNPs in the *APOA5* region, the most significant locus for MetS and two of its diagnostic constituents, plasma triglyceride (TG) and high-density lipoprotein (HDL) levels. Ultimately, the interaction of relevant SNPs with environmental lifestyle factors was analyzed.

## Materials and methods

### Study participants

The Korean Genome and Epidemiology Study (KoGES) is a large prospective cohort study designed to identify genetic and environmental factors and their interactions within common complex diseases such as MetS, osteoporosis, and cancer [16]. The KoGES cohort, in which health examinee participants were followed up from 2007 to 2013, mainly consisted of population-based and gene-environment interaction studies. All ethical considerations and requirements were satisfied, and the need for informed consent was waived because the participants were granted complete anonymity. The dataset used for the current analysis was the KoGES Health Examinee (HEXA) cohort, which mainly consisted of patients recruited from hospitals in major cities in South Korea. Based on the results of the pairwise identity-by-descent (IBD) and multidimensional scaling (MDS) analyses, individuals with familial relationships or those likely to be a cause of population stratification were excluded [17]. All participants were ≥ 40 years of age (starting population 58,700), and 100 participants with unavailable blood test results for TG or unavailable demographics were excluded. Consequently, 58,600 study participants with non-missing demographic variables and available blood test results were included. This study was approved by the Institutional Review Board (IRB) of the Center for Disease Control of the Korean government and the Seoul National University Hospital Clinical Research Institute (IRB No. C-2004-080-1117). The genotyping and phenotyping methods were performed according to the Declaration of Helsinki.

### Phenotype measurements and definition of MetS

Eligible participants completed a questionnaire on demographic factors, comorbidities, and medications for hypertension, diabetes mellitus, and dyslipidemia. Blood tests, including the measurement of fasting blood sugar (FBS), TG, and HDL levels, were conducted using an automated analyzer after 12 h of fasting. MetS cases and controls were ascertained according to the guidelines of the NCEP-ATP III and the American Heart Association/National Heart Lung and Blood Institute (AHA/NHLBI) [18, 19]. The criterion for abdominal obesity was modified according to East Asian guidelines [20]. Patients who met three or more of the following criteria were selected: (1) waist circumference (WC) ≥ 90 cm for males and ≥ 80 cm for females; (2) TG levels ≥ 150 mg/dL; (3) HDL levels ≤ 40 mg/dL in males or ≤ 50 mg/dL in females; (4) blood pressure (BP) levels ≥ 130/85 mmHg or on hypertensive medication; and (5) elevated FBS levels ≥ 100 mg/dL or on medication for diabetes mellitus. Controls without MetS were defined as those who did not satisfy the MetS criteria, or satisfied two or less of the five MetS criteria.

### Ascertainment of covariate variables

Based on their alcohol consumption, the participants were stratified into three groups: non- to low drinkers, moderate drinkers, and heavy drinkers. The dividing criteria for drinking status were assessed based on the frequency of alcohol consumption, volume of alcohol consumed each time, and type of alcohol consumed. Participants who drank ≥ 196 g alcohol per week were defined as heavy drinkers; those who drank 98–196 g of alcohol per week were defined as moderate drinkers; and those who drank < 98 g of alcohol per week were defined as non- to low drinkers. Based on their smoking status, the participants were classified as non- or never-smokers, ex-smokers who quit smoking in the past, or current smokers. Based on frequency and intensity, physical activity was stratified as low (no regular exercise), medium (regular exercise with sweating at a frequency of 1–4 times a week), or high (regular exercise with sweating at a frequency of ≥ 5 times a week).

### Genotyping and imputation

The samples were genotyped using the KoreanChip version 1.1, a customized array optimized for the Korean population. Imputation was performed using the Northeast Asian Reference Database panel v.2. Comprehensive details of the genotyping and genetic imputation methods have been previously described [21, 22]. Finally, 7,857,085 variants with minor allele frequency ≥ 0.01, Hardy–Weinberg equilibrium *P*-value ≥ 1E–06, missing rate < 0.1, and estimated imputation accuracy from Minimac4 (R^2^) ≥ 0.3 were used for the GWAS analysis. The positions of all variants included in this study were based on hg19 reference genome.

### Statistical analysis and visualization of results

Student’s t-test was performed for continuous variables and the chi-square test was performed for categorical variables to compare the groups with and without MetS. For smoking, alcohol consumption, and exercise intensity, the Cochran-Armitage trend test was used to evaluate the *P*-values corresponding to deviations from linear trends. GWAS assessed MetS and its five diagnostic criteria as binary variables. The genetic association between MetS and its five diagnostic criteria, adjusted for age and sex, using logistic regression analysis was implemented via PLINK (version 1.9; Free Software Foundation Inc., Boston, MA, USA). All genotyping data were analyzed using the computing server at the Genomic Medicine Institute Research Service Center. Power analyses and sample size calculations were also performed, in which TG values were calculated for the rs651821 and rs2266788 genotypes. With alpha set to 0.05 and power set to 0.8, the required sample size was 1,158 people, which was well below the population number of the current study or that of any genotype subgroup (minimum subgroup population was 2,857 for rs2266788 GG genotype).

The Genome-wide Complex Trait analysis package, Conditional and Joint association analysis using GWAS summary statistics (GCTA-COJO) [23], was used to extract additional statistically significant and independent variants beyond the most significant variants at each locus contributing to each specific phenotype of BP, WC, FBS, TG, HDL, and MetS. ANNOVAR was used to annotate each variant with information regarding function, neighboring genes, and population frequency [24]. Fine-mapping was performed using POLYgenic FUNctionally informed fine-mapping (PolyFUN), implementing the Sum of Single Effects(SuSiE) statistical model [25]. From these methods, the credible set was restricted to a total of 10 sets for each analysis, and posterior inclusion probability (PIP) values were calculated for each section of ± 100 kilobases for each independent SNP derived from GCTA-COJO. This revealed the PIP values for each SNP. A PIP > 0.9 was defined as a high probability of causality, and a PIP 0.1 cutoff was applied to achieve minimum causality. Fine-mapping was performed for each of the UK Biobank and KoGES city cohorts, with the TG binary variable (cutoff of 150 mg/dL) as the phenotype.

Baseline demographics were analyzed using STATA v.17.0 (StataCorp LLC, College Station, TX, USA). The R package ‘qqman’ was used for the GWAS Manhattan plot and qqplot visualization [26]. The genome-wide significance level for the *P*-value was set at 5E-08. LocusZoom plot was used for magnifying the view of major SNPs of *APOA5* on chromosome 11 [27].

## Results

### Genetic associations from MetS GWAS and its components

**Fig. 1:**
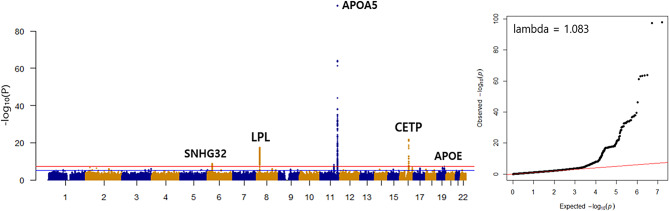
Manhattan and Quantile-Quantile plot of the genome-wide association study of metabolic syndrome(MetS) in Koreans. The x-axis represents the SNP markers on each chromosome. y-axis shows the minus log10 scale. The red horizontal line represents the genome-wide significant threshold *P* = 5E-08 and the blue horizontal line represents the genome-wide suggestive threshold *P* = 1E-05. Five candidate loci of MetS are shown

Among the 58,600 KoGES study population, approximately 24% of the total study population satisfied 3 or more out of the total 5 MetS diagnostic criteria. Age, sex, body mass index, and lifestyle factors such as smoking, drinking, and physical activity differed significantly between the patients with and without MetS (Table 1). GWAS and conditional and joint analysis (COJO) for MetS revealed 11 independent signals: *APOA5* on chromosome 11 was the most significant signal (rs651821, odds ratio [OR] = 1.37, *P*-value = 1.25E-98), which was followed by *CETP* on chromosome 16 (rs17231506, OR = 0.83, *P*-value = 2.57E-23), and *LPL* on chromosome 8 (rs59147390, OR = 0.83, *P*-value = 1.00E-18) (Fig. 1; Table 2). GWAS and COJO performed on each of the five MetS diagnostic criteria (Figure S1, Table S1) showed that the number of independent signals that reached a genome-wide significance level was 16 for BP, four for WC, 25 for FBS, 18 for TG, 34 for HDL (a total of 97 SNPs for MetS diagnostic components) (Table S1). *APOA5*,* BUD13*, *LPL*, and *APOE* loci were all associated with MetS/TG/HDL (Figure S2). These genes can be functionally explained or have been identified in GWAS related to lipid metabolism [28–31]. Linkage disequilibrium score (LDSC) was also calculated for MetS GWAS. The genomic inflation factor (λGC) was 1.083 (Fig. 1), LDSC intercept was 1.010 (standard error (SE) = 0.004), LDSC ratio was 0.099 (SE = 0.042); implying that polygenicity, but not confounding factors, such as population stratification or cryptic relatedness, was the principal cause of genomic inflation [32]. In addition to age/sex adjustment, further adjustment of lifestyle factors(smoking, alcohol drinking, physical activity) and 5 genetic principal components in GWAS analyses revealed minute differences in the results (Table S2).

**Table 1:** Baseline characteristics of the study population (*N* = 58,600)

Characteristics	Case with MetS	Control	*P*-value
	Mean ± SD or number (%)	Mean ± SD or number (%)	
Total number	14,323 (24.4%)	44,277 (75.6%)	
Age (years)	56.4 ± 7.6	53.0 ± 8.0	< 0.001
Male number (%)	5,538 (38.7%)	14,738 (33.3%)	< 0.001
BMI (kg/m^2^)	25.9 ± 2.8	23.2 ± 2.6	< 0.001
**MetS Components**			
WC (cm)	87.2 ± 7.4	78.7 ± 7.9	< 0.001
HDL Cholesterol (mg/dL)	45.0 ± 10.0	56.6 ± 12.8	< 0.001
TG (mg/dL)	193.1 ± 115.2	103.1 ± 58.5	< 0.001
Fasting blood sugar (mg/dL)	106.4 ± 27.6	91.4 ± 14.5	< 0.001
Systolic BP (mmHg)	130.9 ± 14.1	119.7 ± 13.9	< 0.001
Diastolic BP (mmHg)	80.3 ± 9.4	74.3 ± 9.4	< 0.001
**Health related behaviors**			
Smoking			< 0.001
Non-smoker	3,126 (69.6%)	10,127 (75.7%)	
Ex-smoker	784 (17.5%)	1,917 (14.3%)	
Current smoker	581 (12.9%)	1,331 (10.0%)	
Drinking			0.004
Non to low drinker	7,593 (53.9%)	22,713 (52.1%)	
Moderate drinker	4,375 (31.0%)	15,945 (36.6%)	
Heavy drinker	2,124 (15.1%)	4,936 (11.3%)	
Physical activity category			< 0.001
Low level	6,816 (48.6%)	19,708 (45.5%)	
Moderate level	4,347 (31.0%)	14,771 (34.1%)	
High level	2,856 (20.4%)	8,864 (20.4%)	

BMI: Body mass index; BP: Blood pressure; HDL: High density lipoprotein; MetS: Metabolic syndrome; SD, standard deviation; TG: Triglycerides; WC: Waist circumference

Student’s t-test was done for continuous variables and chi-square test for categorical variable. Cochran-Armitage test for trend was done for ordered categorical variables in order to compare the groups with and without MetS

**Table 2:** MetS associated independent SNPs in the KoGES cohort grouped by each locus

SNP	CHR:POS (hg19)	Effect/N-E allele	EAF (case/control)	*P*-value	OR (95% CI)	Nearest Gene(s)	Functional Consequence
rs651821	11:116662579	C/T	0.347/0.282	1.25E-98	1.37 (1.33–1.41)	*APOA5*	5’UTR
rs74368849^a^	11:116622299	A/G	0.107/0.076	2.89E-18	1.10 (1.08–1.13)	*BUD13*	intronic
rs141740187^a^	11:116637677	T/G	0.207/0.209	3.50E-09	1.05 (1.03–1.06)	*BUD13*	intronic
rs17231506	16:56994528	T/C	0.154/0.179	2.57E-23	0.83 (0.80–0.86)	*HERPUD1;CETP*	intergenic
rs2303790^a^	16:57017292	G/A	0.038/0.047	2.00E-08	0.92 (0.89–0.94)	*CETP*	exonic (p.D459G)
rs59147390	8:19843879	C/T	0.110/0.129	1.00E-18	0.83 (0.79–0.86)	*LPL;SLC18A1*	intergenic
rs6586892^a^	8:19941145	A/C	0.199/0.210	1.58E-08	0.96 (0.94–0.97)	*LPL;SLC18A1*	intergenic
rs112405902	6:31809504	A/G	0.080/0.069	4.49E-10	1.18 (1.12–1.24)	*SNHG32;NEU1*	intergenic
rs429358	19:45411941	C/T	0.101/0.090	8.62E-10	1.15 (1.10–1.21)	*APOE*	exonic (p.C130R)
rs10830963	11:92708710	G/C	0.446/0.426	9.47E-09	1.08 (1.05–1.11)	*MTNR1B*	intronic
rs731839	19:33899065	A/G	0.454/0.471	2.50E-08	0.93 (0.90–0.96)	*PEPD*	intronic

CHR: Chromosome; CI: confidence interval; EAF: Effect allele frequency; KoGES: Korean Genome and Epidemiology Study cohort; MetS: Metabolic syndrome; N-E allele: Non-effect allele; OR: Odds ratio; POS: Position; SNP: Single nucleotide polymorphism; UTR: untranslated region

SNPs are in order of increasing *P*-values for each loci and for each top signal SNP

^a^Joint analysis results derived from genome-wide complex trait conditional and joint(GCTA-COJO) multiple SNP analyses

Based on the COJO-derived variants from GWAS of MetS components, 12 exonic and non-synonymous SNPs were identified (Table S3). For the 11 independent signals derived from MetS GWAS (Table 2), the genetic association of each variant for each of the five MetS components was further illustrated (Figure S3, Table S4). The most significant *APOA5* locus in the MetS GWAS was also the most significant in TG and HDL GWAS (Figure S4). Although differences in the magnitude of statistical significance were noticed, HDL and TG levels overlapped most with the results of MetS GWAS, thereby corroborating the findings of a previous study indicating that lipid traits considerably contribute to the MetS phenotype [33]. In addition, among the 11 MetS signals, one overlapped with BP (*SNHG32*), three overlapped with WC (*SNHG32*,* MTNR1B*,* PEPD*), and two overlapped with FBS (*MTNR1B*,* PEPD*), all with the same OR valence except for rs731849 (*PEPD*) in WC.

### Relationship between *APOA5* variants and lipid levels

Regarding the *APOA5* signal, which was most significant for MetS, TG, and HDL, rs651821 was the most significant variant for MetS and TG, whereas rs662799 was the most significant variant for HDL, with an LD (*r*^*2*^) > 0.99 between the two variants (Figure S4). In addition to rs651821 and rs662799, there were other significant variants within the *APOA5* locus, including rs2072560 and rs2266788 (Table 3). The relationship between these variants and TG/HDL levels was also analyzed; when these variants were adjusted for rs651821, their OR directions were reversed for both lipid traits. This particular multilayered phenomenon was further replicated in a separate KoGES rural cohort (*N* = 8,102), where the OR for rs2266788 was reversed when conditioned with the top rs651821 SNP (rs2266788 unconditioned OR/beta/*P*-value 1.38/0.14/2.44E-16, rs651821 conditioned rs2266788 OR/beta/*P*-value 0.78/-0.11/1.63E-04). The LD (*r*^*2*^) between rs651821-rs2072560 and rs651821-rs2266788 was 0.65 each, whereas rs2072560 and rs2266788 were highly correlated with each other(*r*^*2*^ = 0.995). The *P*-value of epistatic interaction for rs651821-rs2266788 SNPs was 0.067, thereby excluding the possibility of SNP-SNP pairwise epistatic effects. rs2072560 is located in the intron of *APOA5*, whereas rs2266788 is located in the 3’-untranslated region(UTR) of *APOA5*; rs2266788 has been reported in ClinVar [34] as a potential risk variant for familial hypertriglyceridemia [35–37] (ClinVar Accession number SCV000148905).

**Table 3:** GWAS and conditioned results of *APOA5* major SNPs on TG and HDL

SNP	Effect allele	CHR: POS (hg19)	EAF	Functional consequence	* LD (*r*^2^)	TG GWAS		TG rs651821 Conditioned		HDL GWAS		HDL rs651821 Conditioned	
						*P*-value	OR	*P*-value	OR	*P*-value	OR	*P*-value	OR
rs651821	C	11:116662579	0.259	5’-UTR	-	3.69E-296	1.72	-	-	1.71E-180	1.50	-	-
rs662799	G	11:116663707	0.298	Intergenic	0.995	1.95E-293	1.71	0.31	0.78	1.09E-180	1.50	0.14	1.32
rs2072560	T	11:116661826	0.235	Intronic	0.654	1.76E-119	1.45	2.87E-27	0.76	2.53E-46	1.25	6.39E-49	0.69
rs2266788	G	11:116660686	0.218	3’-UTR	0.653	3.99E-118	1.45	4.36E-28	0.75	1.41E-45	1.24	6.68E-50	0.69

CHR: Chromosome; EAF: Effect allele frequency; HDL: High density lipoprotein; LD: Linkage Disequilibrium; OR: Odds ratio; POS: Position; SNP: Single Nucleotide Polymorphism; TG: Triglycerides; UTR: Untranslated region

* LD in Korean population with rs651821 of each SNP is specified (excluding rs651821 itself)

### Inter-population differences in genetic associations and fine-mapping of KoGES and UK Biobank cohorts

Within the 11 COJO-derived independent signals of the MetS GWAS, no cases were observed where signals remained significant but had reversed ORs after conditioning the top SNPs of each locus, except for rs651821. Within the UK Biobank cohort, conditional analyses of rs651821 and rs2266788 revealed results distinct from those of KoGES, where OR directions were maintained even after conditional analysis. To determine the mechanisms underlying these different results across populations, fine-mapping of each cohort by classifying plasma triglyceride values as a binary variable (150 mg/dL according to the MetS criteria) was performed which revealed distinct characteristics because no overlapping SNPs were observed among SNPs with PIP values > 0.9 (Figure S5). In the KoGES cohort, rs651821 located in *APOA5* locus on chromosome 11 was an independent SNP and potential causal variant in Koreans, with a PIP value of 0.998 (Table S5). However, when the same analysis was performed using UK Biobank data, rs964184, located at the 3’-UTR of *ZPR1*, was identified as the top SNP (Figure S6) with a high probability of causality (Table S6) whereas rs651821 had a PIP value of approximately zero and was not included in the ten derived credible sets. Nevertheless, rs4938311 located in *BUD13*, was the only overlapping SNP with a PIP > 0.1 in both cohorts. Further, LD blocks of KoGES and UK Biobank cohorts at the *APOA5* locus showed the inter-population differences in genetic architecture (Figure S7).

### Association between genotypes of rs651821 and rs2266788 with the prevalence of MetS, and serum TG and HDL levels

**Fig. 2:**
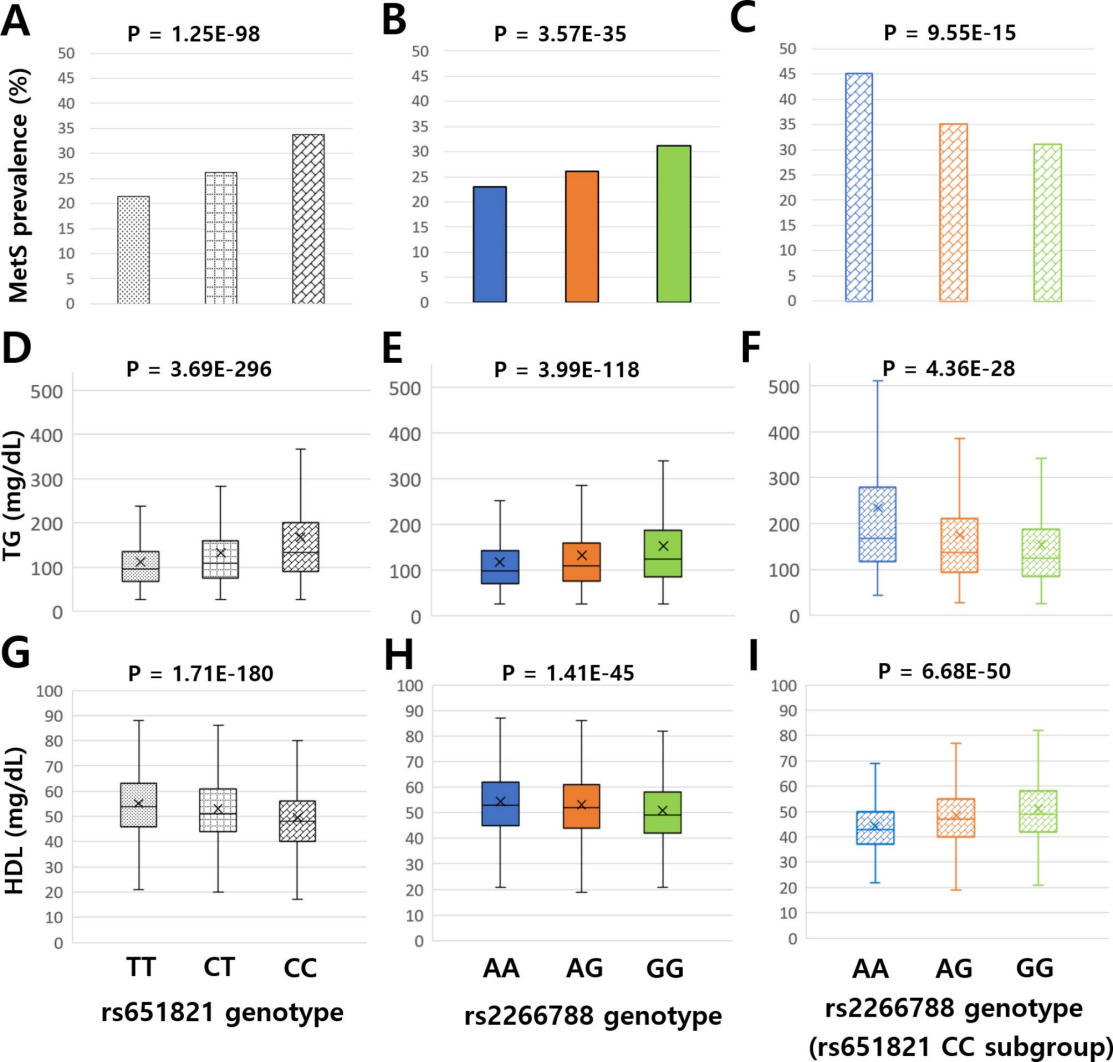
Multilayered effects of rs651821 and rs2266788 in MetS prevalence, TG and HDL. Metabolic syndrome prevalence percentage (**A-C**), plasma TG (**D-F**) and plasma HDL (**G-I**) levels according to rs651821 and rs2266788 genotypes. Corresponding values according to rs651821 genotypes (**A**,** D**,** G**), rs2266788 genotypes (**B**,** E**,** H**), rs2266788 genotypes within rs651821 CC subgroup (**C**,** F**,** I**) are depicted.

The prevalence of MetS were 21%, 26%, and 34% for TT, CT, and CC genotypes, respectively, within rs651821 (Fig. 2A, Table S7). MetS prevalence was 23%, 26%, and 31% for AA, AG, and GG genotypes, respectively, within rs2266788 (Fig. 2B). However, within the rs651821 CC subgroup, MetS prevalence were 45%, 35%, and 31% for AA, AG, and GG genotypes, respectively, within rs2266788, indicating a multilayered effect between rs651821 and rs2266788 (Fig. 2C). Serum TG levels, according to the rs651821 genotype, increased as the minor allele (C allele) count increased (Fig. 2D). Similarly, serum TG levels, according to the rs2266788 genotype, significantly increased as the minor allele (G allele) count increased (Fig. 2E). In contrast, in the rs651821 CC genotype subgroup (homozygous minor allele subgroup), serum TG levels decreased as the minor allele count of rs2266788 increased (Fig. 2F). Similarly, the HDL levels decreased when the minor allele count of rs651821 increased (from TT to CT to CC) (Fig. 2G) or when the minor allele count of rs2266788 increased (from AA to AG to GG) (Fig. 2H). However, within the rs651821 CC subgroup, the HDL levels significantly increased when the minor allele count of rs2266788 increased (from AA to AG to GG) (Fig. 2I).

### Gene-environment interactions in metabolic factors

**Fig. 3:**
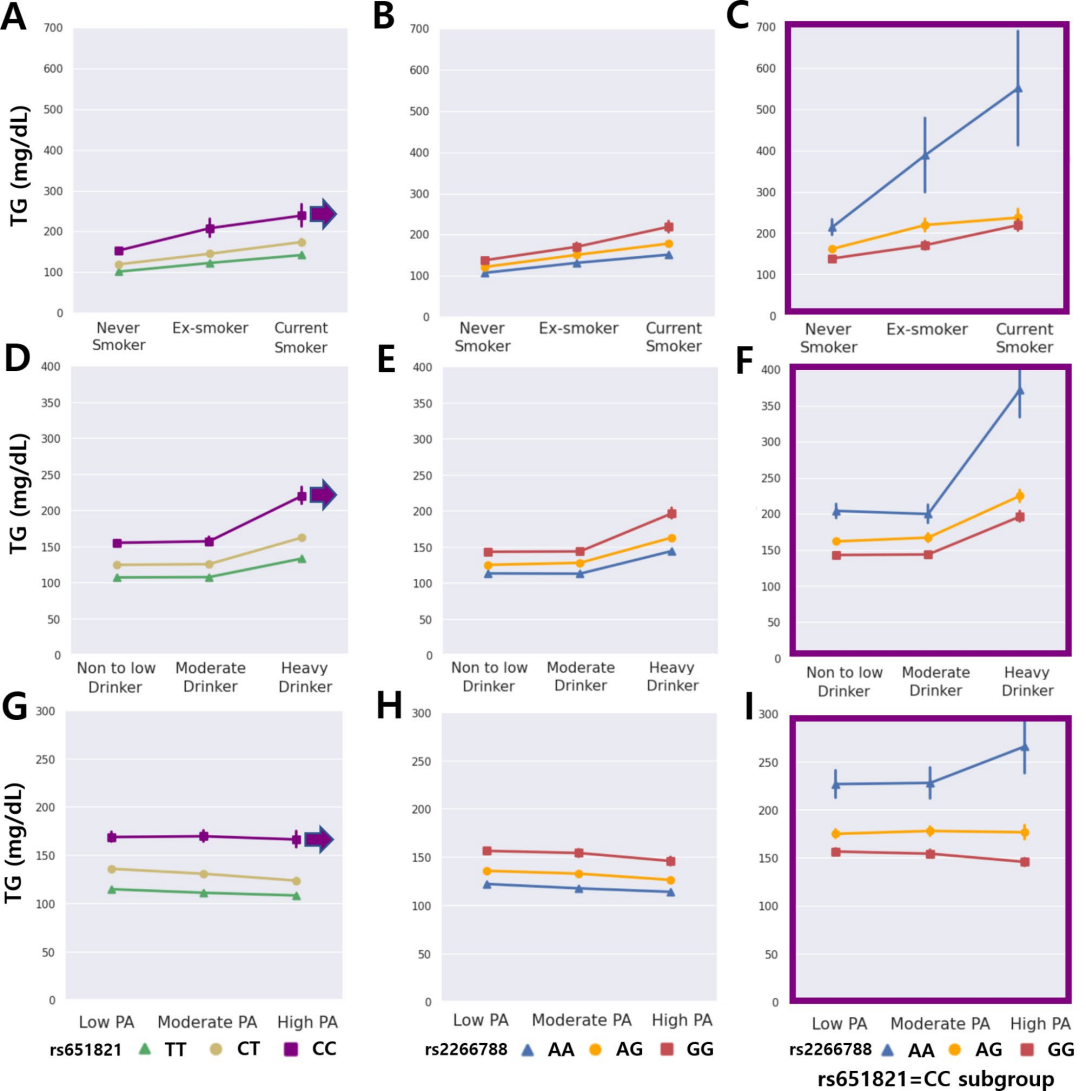
Effects of multilayered SNPs in MetS component TG levels and their interactions with lifestyle factors. TG levels according to rs651821, rs2266788 genotypes associated with stratified levels of smoking (**A-C**), alcohol drinking (**D-F**) and physical activity (**G-I**). The purple arrows referring to the boxes (**C**,** F**,** I**) indicate minor homozygous subgroup of rs651821 CC genotype. All error bars represent standard errors. PA: physical activity

Because metabolic factors are influenced by genetic and environmental factors, three representative lifestyle environmental factors, such as smoking, alcohol consumption, and physical activity, were examined to evaluate gene-environment interactions. Regarding smoking habits, TG levels were lowest in non-smokers, followed by ex-smokers and current smokers. When stratified into genotypes, the minor homozygous rs651821 CC subgroup and rs2266788 GG subgroup were associated with the highest TG levels (Fig. 3A and B). However, within the rs651821 CC subgroup, the rs2266788 AA subgroup was associated with the highest TG levels, which were all higher than those of the rs2266788 GG subgroup (Fig. 3C). The overall trend of TG levels increased from moderate to heavy drinkers; however, the rs2266788 correlation of AA-AG-GG was reversed in the rs651821 CC genotype subgroup (Fig. 3D, E and F). This reversal of TG levels for the rs651821 CC subgroup is in line with data presented in Fig. 2, except that the additional analysis of environmental factors strengthened the putative hypothesis. Individuals with rs651821 CC and rs2266788 AA genotypes have significantly elevated TG levels when smoking (Fig. 3C) or excessively drinking (Fig. 3F), but with highly relatively highly increasing TG slopes compared to those of the rs651821 genotypes analyzed as a whole (Fig. 3B and E). Further, within the rs651821 CC subgroup, TG levels of the rs2266788 AA subgroup increased when the extent of physical activity increased (Fig. 3I). HDL levels were also analyzed for gene-environment interactions and showed similar results regarding rs651821 and rs2266788 with respect to lifestyle factors (Figure S8). Particularly, regarding physical activity, ‘beneficial’ HDL cholesterol levels increased with increasing extents of physical activity within rs651821 (Figure S8G) and rs2266788 (Figure S8H) genotypes analyzed as a whole, specifically conspicuous within the rs2266788 AA subgroup. However, within the rs651821 CC and rs2266788 AA subgroups (Figure S8I), high extent of physical activity conversely reduced HDL cholesterol levels, suggesting an interaction between genetic and environmental factors in combination with the multilayered effects.

## Discussion

In this study, MetS GWAS of 58,600 Koreans from the KoGES database was performed and 11 independent signals with genome-wide significance were identified. Additional analysis uncovered a complex relationship between the two SNPs (rs651821 and rs2266788), which was not apparent in a single GWAS, and revealed the actual direction of the effect of rs2266788 on MetS and lipid traits. These two SNPs located within the *APOA5* locus, each 5’-UTR and 3’-UTR variants respectively, have been identified as possible controllers of fasting and postprandial TG levels and recently came into the spotlight in genetic studies involving humans and mice [38]. In addition, this study considered environmental factors such as alcohol consumption, smoking status, and exercise frequency and assessed the relationship between these factors and different genotypes. Previously, MetS GWAS has been performed on the Korean population [39, 40]. Although these studies uncovered previously unidentified SNPs, they had a limited sample size of a few thousand individuals, resulting in limited study scope. Furthermore, a previous study [41] has also investigated the interaction between *APOA5* rs662799 and rs2266788, which collectively influenced TG concentrations, and lifestyle factors such as carbohydrates, calcium intake, and smoking; however, they did not explore the intricate relationship between the two SNPs.

The *APOA5* locus located in chromosome 11 was the most significant loci for all MetS, TG, and HDL GWAS. The underlying mechanism, through which increased *APOA5* expression leads to decreased TG levels has been previously described [36, 42–44]. Regarding *apoav*, which is the mouse equivalent of *APOA5*, transgenic knockout mice have shown 4-fold higher TG levels than those in control mice, whereas *APOA5*-overexpressing mice had one-third lower TG concentrations than those of the controls [36]. The *apoav* lipid complexes bind to heparin, thereby indirectly affecting lipoprotein lipase (LPL) activity in vitro [44]. *APOA5* stimulates the LPL-mediated hydrolysis of lipoproteins, for instance very low-density lipoprotein (VLDL) and chylomicrons, which are inhibited by *APOE* [43]. A study using immunoprecipitation and mass spectrometry reported that *APOA5* lowers TG levels by suppressing ANGPTL3/8-mediated inhibition of LPL in the human serum [42].

In addition to *APOA5*, GWAS on MetS and its diagnostic components revealed numerous significant signals associated with two or more MetS components that should be functionally mentioned. *CETP*, associated with MetS/HDL, encodes a cholesteryl ester transfer protein that transfers cholesteryl ester and TG between HDL, LDL, and VLDL [45]. Furthermore, rs10830963 located within *MTNR1B*, a gene encoding melatonin receptor 1B, has been associated with MetS/FBS [46]. MTNR1B is related to circadian rhythms and is implicated in mediating the inhibitory effect of melatonin on insulin secretion. *BRAP*, associated with HDL/FBS, encodes the breast cancer suppressor protein-associated protein (BRAP) and is associated with obesity and metabolic factors in an East Asian population [47]. GCKR is highly associated with TG among MetS components [48], which is in line with the current study’s conclusion regarding the association of *GCKR* with TG/FBS levels. Increased activity of the *GCKR* influences glucokinase to regulate the first step of glycolysis. Glycolysis provides acetyl-CoA intermediates, thereby fueling the synthesis of fatty acids, which leads to increased lipogenesis in the liver and, in this case, TG [49].

Although rs651821 and rs2266788 were both identified in previous TG GWAS, rs651821 has received much attention owing to its high significance. GATA4 inclines to bind to the protective T allele of rs651821, thereby increasing *APOA5* expression in liver cells and in individuals with the TT genotype, which ultimately leads to decreased TG levels [50]. Each genomic variant may affect the expression of a certain gene through the enhancer/promoter activities or mRNA stability to influence a certain phenotype [51]. Whether rs2266788 had enhancer activity towards *APOA5* remained controversial [52]. Cui et al. [53] have reported that the G allele of this 3’-UTR variant of *APOA5* increases *APOA5* expression by maintaining more stability of APOA5 mRNA than does the A allele. Alternatively, the A allele of rs2266788 enhanced the degradation of *APOA5* mRNA, and *APOA5* mRNA with the G allele has a longer half-life than that with the A allele. Therefore, these results concur with those of the present study, rendering the G allele of rs2266788 protective towards both MetS and lipid levels. Based on these insights, both variants rs651821 and rs2266788 appear to be functional and to independently contribute to MetS.

### Strengths

This is the first report of multilayered effects of rs651821 and rs2266788, SNPs of *APOA5*, regarding MetS, TG and HDL. Additionally, we identified ethnic differences of the *APOA5* region. To elaborate, comparing the *APOA5* locus for TG levels in the UK Biobank cohort, we found that the most significantly associated SNP and overall genetic structure were different between populations. Furthermore, we performed gene-environment interaction analysis of lifestyle factors, such as smoking, alcohol consumption, and physical activity, and identified that multilayered effects were relatively strong in current smokers and heavy drinkers. These results suggest that the risk assessment of metabolic disease development should be tailored according to personal genotypes and lifestyle factors.

### Limitations

The limitation of this study is that although GWAS on MetS and its components was performed, previously undiscovered loci could not be identified. Instead, this study focused on the most significant loci of MetS, TG, and HDL and identified the multilayered effect of two SNPs (rs651821 and rs2266788). Furthermore, the gene-environment interaction analysis performed herein reports observational characteristics; therefore establishing causal relationships is difficult, and accuracy issues remain owing to the self-reported nature of the three main lifestyle factors. Other environmental factors such as dietary habits or sleep patterns should also be explored in future studies. Last, functional validation studies, such as allele-specific differences in *APOA5* expression for relevant cell types and tissues and in vivo experiments using mouse models associated with MetS, are necessary to support the results presented herein.

## Conclusions

GWAS on MetS and its components revealed the most influential SNPs regarding MetS and its components. The effect of *APOA5* SNPs rs651821 and rs2266788 on MetS and serum lipid levels was analyzed, and gene-environment interaction observations underscored the multilayered effects between the *APOA5* SNPs. From the perspective of precision medicine, personalized genotyping and counseling of the general population could be beneficial and could inform patients how to enhance lifestyle factors and ultimately prevent or overcome MetS.

### Electronic supplementary material

Below is the link to the electronic supplementary material.

Supplementary Material 1: Supplementary tables

Supplementary Material 2: Supplementary figures

## Data Availability

This study was conducted using bioresources from the National Biobank of Korea and the Centers for Disease Control and Prevention Agency (KDCA), Republic of Korea (KBN-2020-029). The KoGES data utilized for this study are not available to the public, but can be requested from the KDCA website www.kdca.go.kr with an appropriate research application form and separate institutional review board consent for the study. This research has also been conducted using the UK Biobank Resource under Application Number 97642.

## Abbreviations

*APOA5*: Apolipoprotein A5
BMI: Body mass index
BP: Blood pressure
FBS: Fasting blood sugar
GCTA: COJO-Genome-wide complex trait analysis-conditional and Joint multiple SNP association analysis
GWAS: Genome-wide association study
HDL: High-density lipoprotein cholesterol
HEXA: Health examinee study
KoGES: Korean genome and epidemiology study
LD: Linkage disequilibrium
MetS: Metabolic syndrome
PIP: Posterior inclusion probability
SNP: Single nucleotide polymorphism
TG: Triglycerides
UTR: Untranslated region
WC: Waist circumference
