# Supplementary material for: Genome-wide association study for metabolic syndrome reveals APOA5 single nucleotide polymorphisms with multilayered effects in Koreans
Source: Lipids Health Dis. 2024 Aug 28;23:272. doi: 10.1186/s12944-024-02248-0 (PMC11351254; doi:10.1186/s12944-024-02248-0)
Supplement: Supplementary file 2 — Supplementary Material 2: Supplementary figures [file 12944_2024_2248_MOESM2_ESM.docx]

**Supplementary Figures Overview**

**Figure S1.** Manhattan plots of GWAS of five MetS components

**Figure S2.** UpSet plot of signals related to MetS and its components

**Figure S3.** MetS associated GCTA-COJO SNPs in relation with each MetS component

**Figure S4.** Locus zoom plots including *APOA5* SNPs within MetS/TG/HDL phenotypes

**Figure S5.** Comparison of fine-mapping results from KoGES and UK Biobank cohorts of TG values

**Figure S6.** Regional association plots for *APOA5* locus in TG GWAS regarding KoGES and UK Biobank cohorts

**Figure S7.** LD Blocks of the KoGES and UK Biobank cohorts

**Figure S8.** Multilayered effects of rs651821/rs2266788 in interaction with HDL and environmental factors

**Figure S1.** Manhattan plots of GWAS of five MetS components


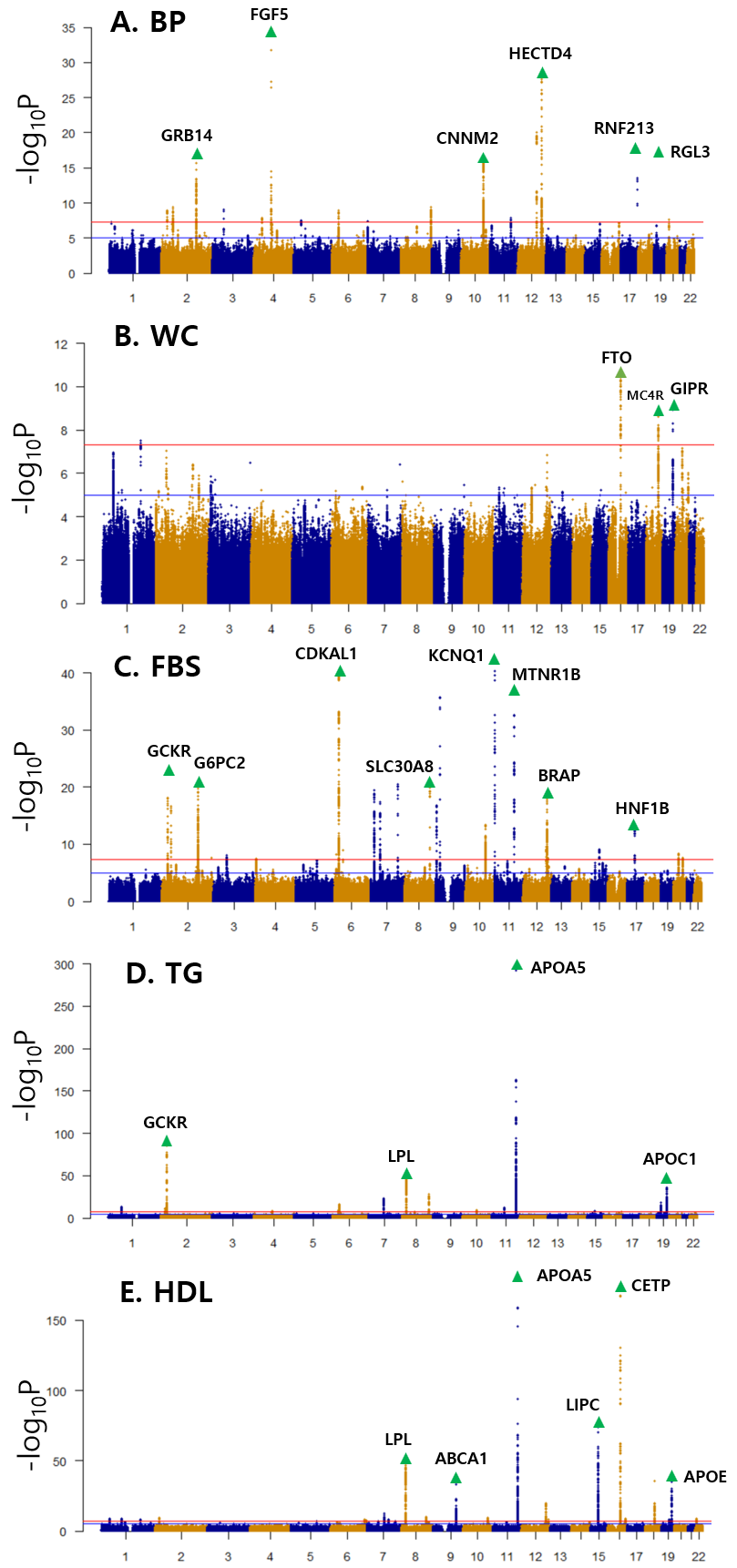


BP: Blood pressure, WC: Waist circumference, FBS: Fasting blood sugar, TG: Triglycerides, HDL: High-density lipoprotein cholesterol

**Figure S2.** UpSet plot of signals related to MetS and its components


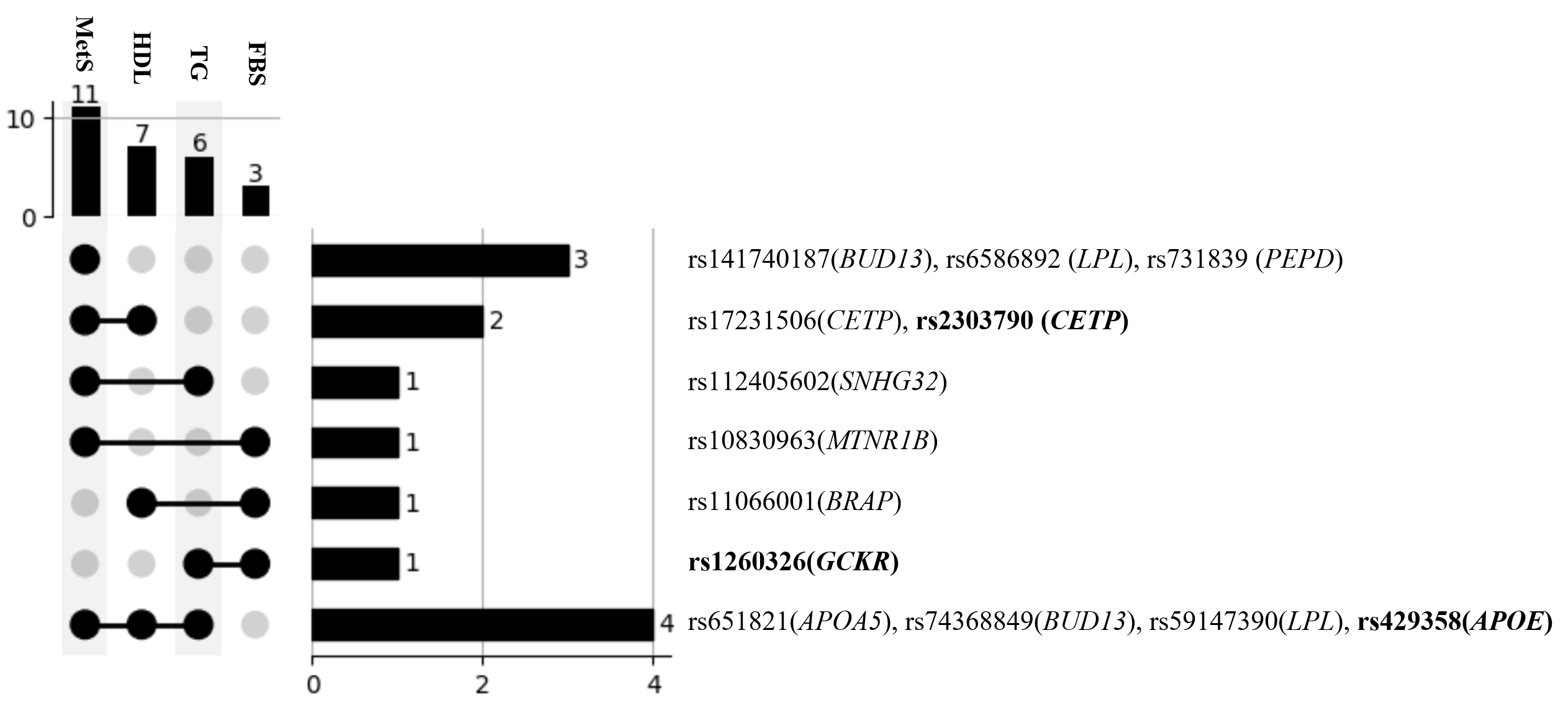


SNPs related to MetS and its components. The left upset plot indicates if each MetS GCTA-COJO(Genome-wide complex trait analysis related conditional and joint multiple-SNP analysis) derived SNP is associated with each MetS component with genome-wide significance (P-value<5E-08). The horizontal bar graph and its number indicate how many SNPs are associated with corresponding traits, and each SNP rsID and its nearest gene are indicated accordingly to the right. Bold indicates non-synonymous exonic SNPs.

**Figure S3.** MetS associated GCTA-COJO SNPs in relation with each MetS component


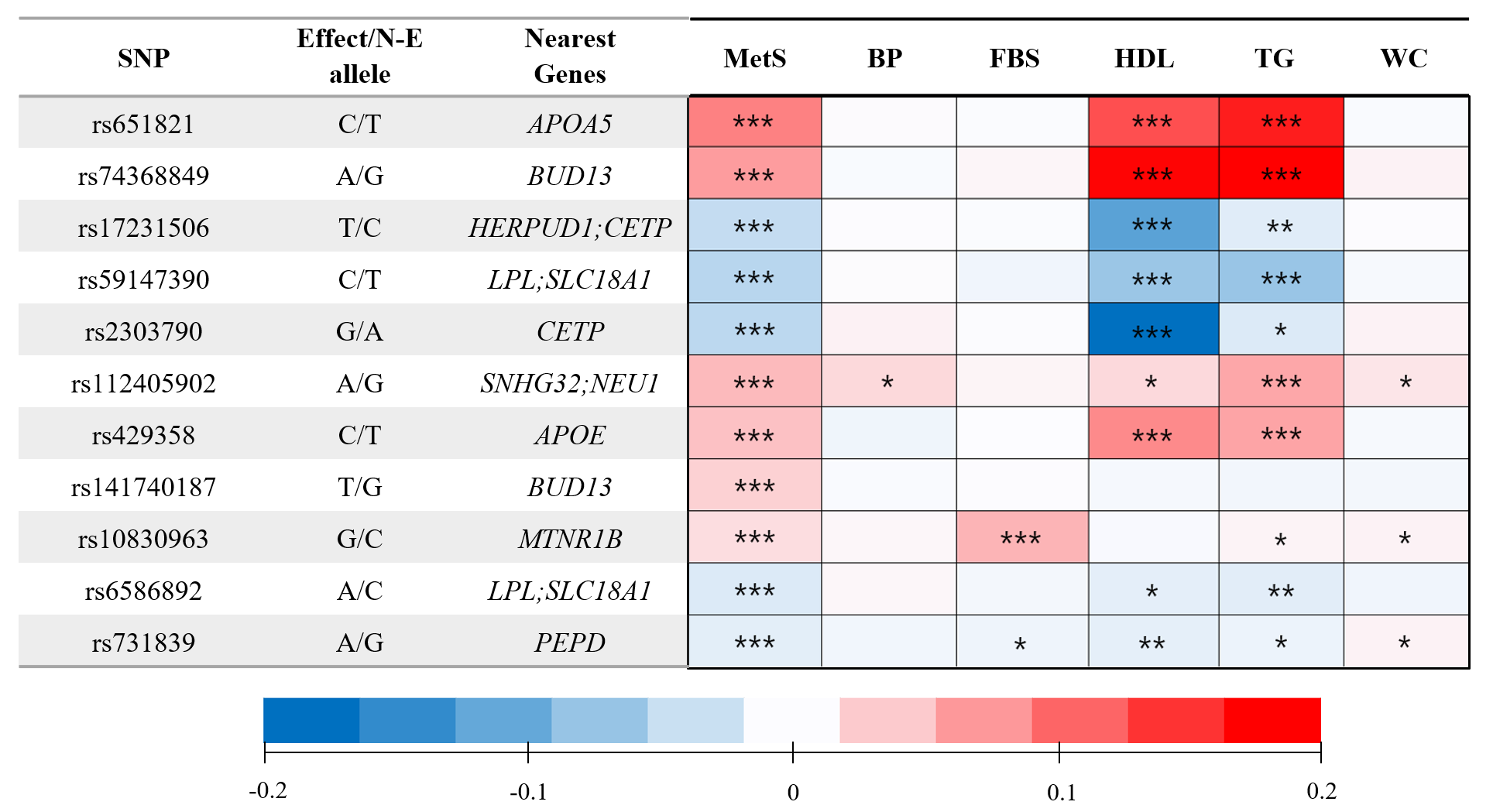


Colors refer to the size and direction of the beta effect.

Asterisks indicate *P*-value significance (*** < 5E-08 , ** < 1E-05 , * < 0.05)

GCTA-COJO: Genome-wide complex trait analysis - Conditional and Joint multiple SNP analysis, SNP: Single Nucleotide Polymorphism, BP: Blood pressure, FBS: Fasting blood sugar, HDL: High-density lipoprotein, TG: Triglyceride, WC: Waist circumference, MetS: Metabolic syndrome, N-E allele: Non-effect allele

**Figure S4.** Locus zoom plots including *APOA5* SNPs within **A.** MetS, **B.** TG and **C.** HDL


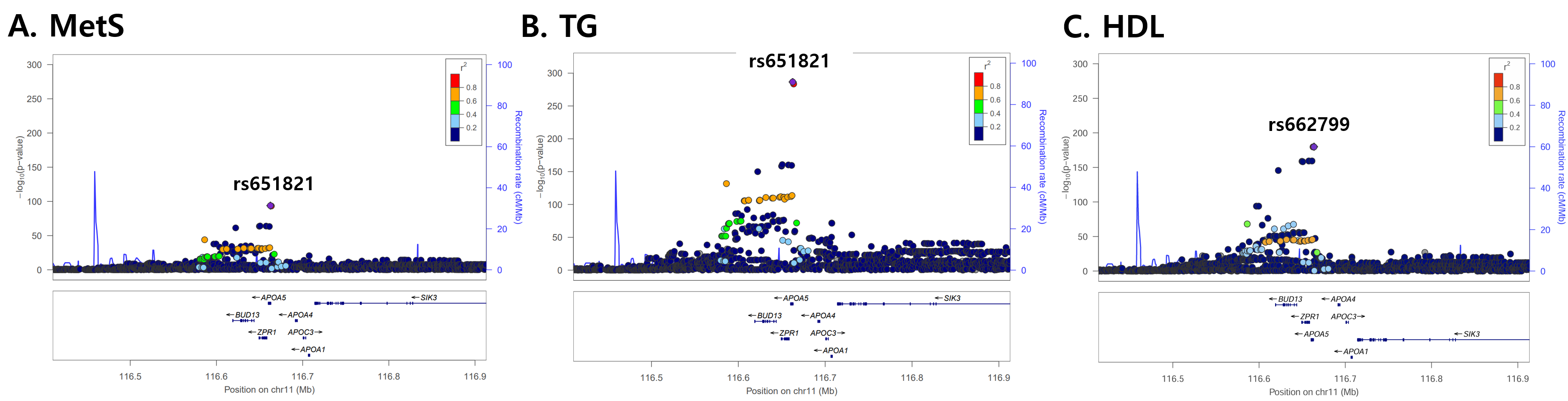


**Figure S5.** Comparison of fine-mapping results from KoGES and UK Biobank cohorts of TG values


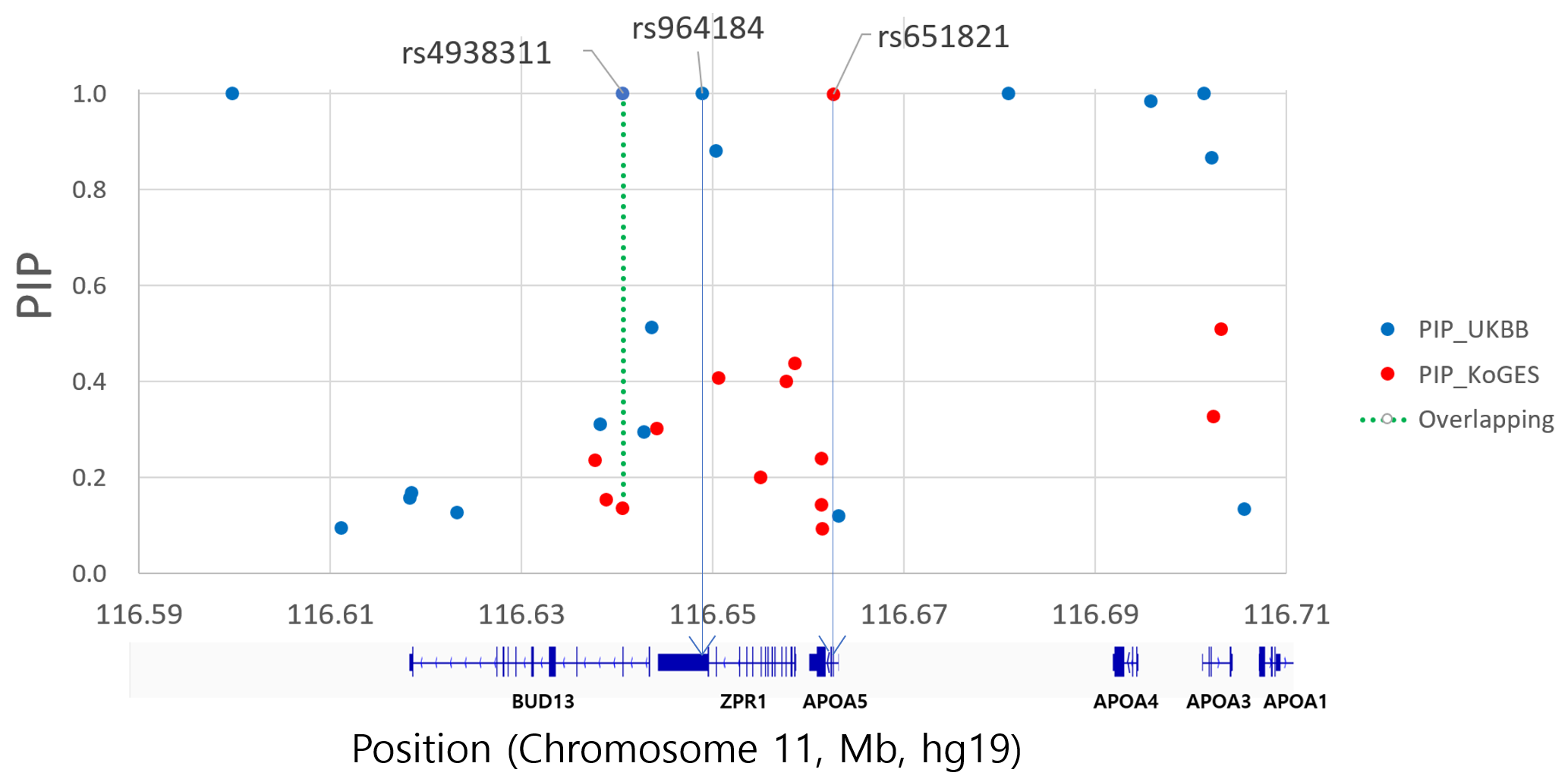


The x-axis shows the genetic position(hg19) on the chromosome 11 *APOA5* region and y-axis shows the posterior inclusion probability (PIP). The red and blue circles represent the SNPs of Koreans and UK Biobank. Dotted line indicates overlapping SNPs. SNPs of PIPs over 0.1 are depicted.

**Figure S6.** Regional association plots for *APOA5* locus in TG GWAS regarding KoGES and UK Biobank cohorts

**
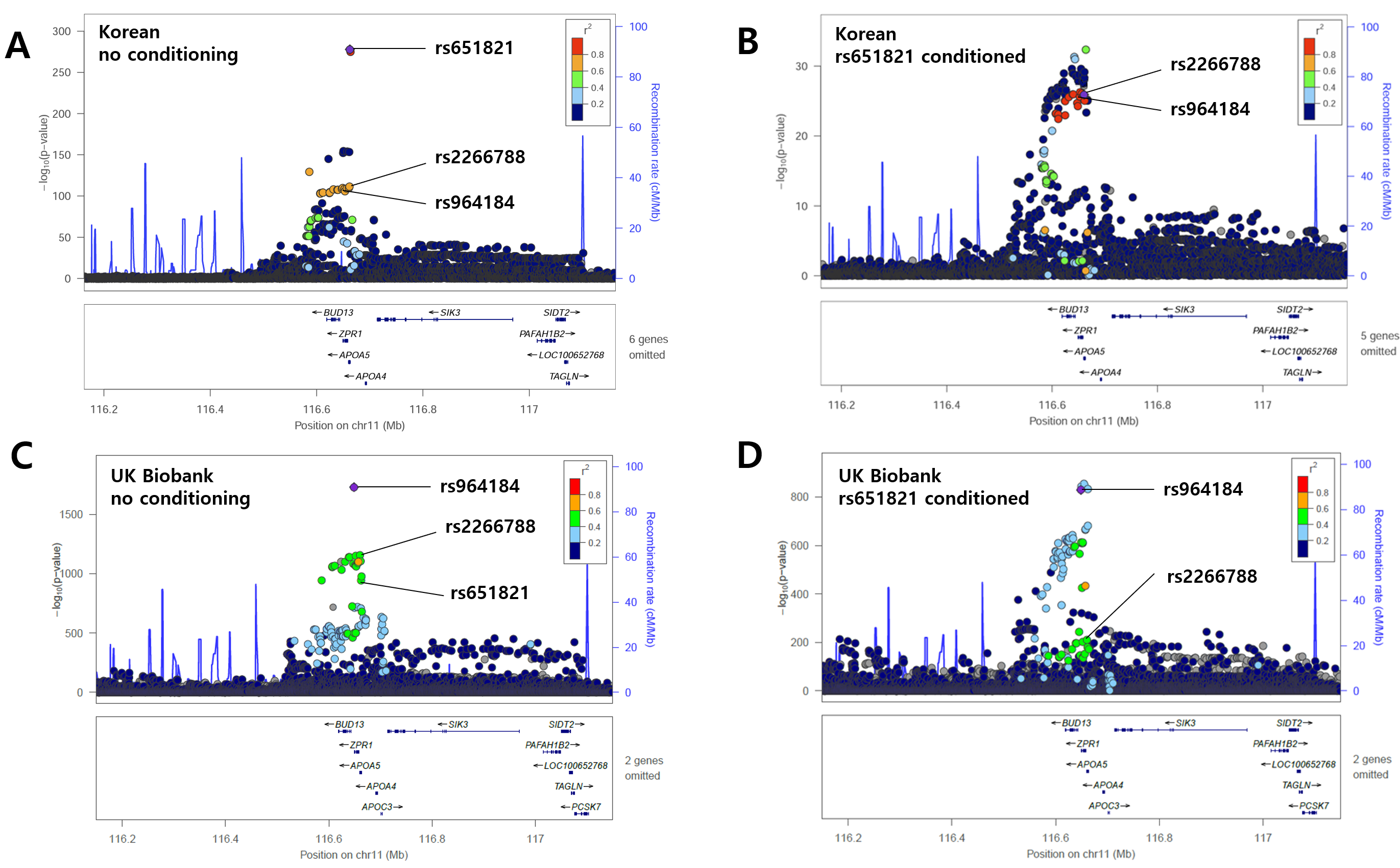
**

**Figure S7.** LD Blocks of the **(A)**KoGES and **(B)**UK Biobank cohorts derived from separate TG GWAS

**
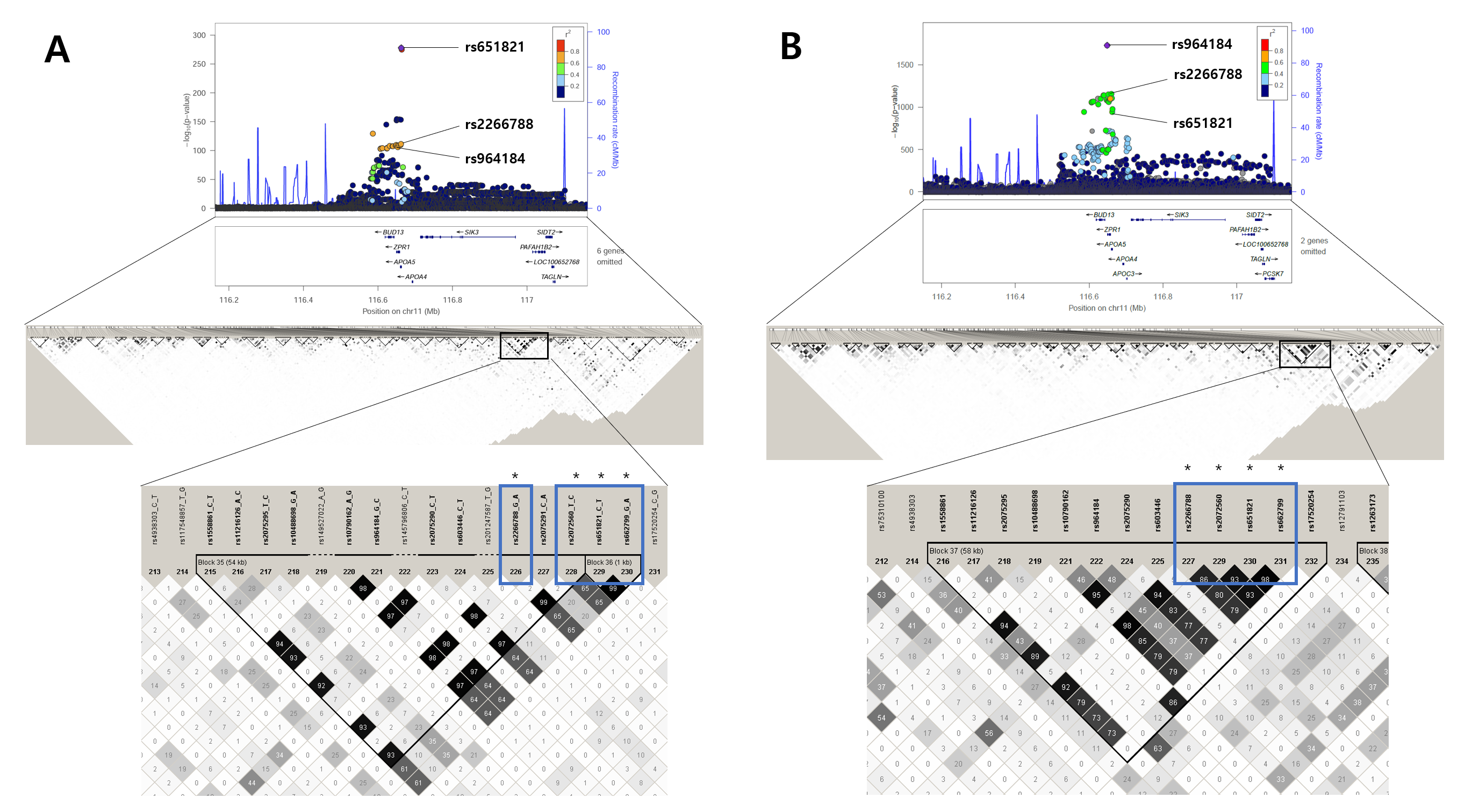
**

**Figure S8.** Multilayered effects of rs651821/rs2266788 in interaction with HDL and environmental factors


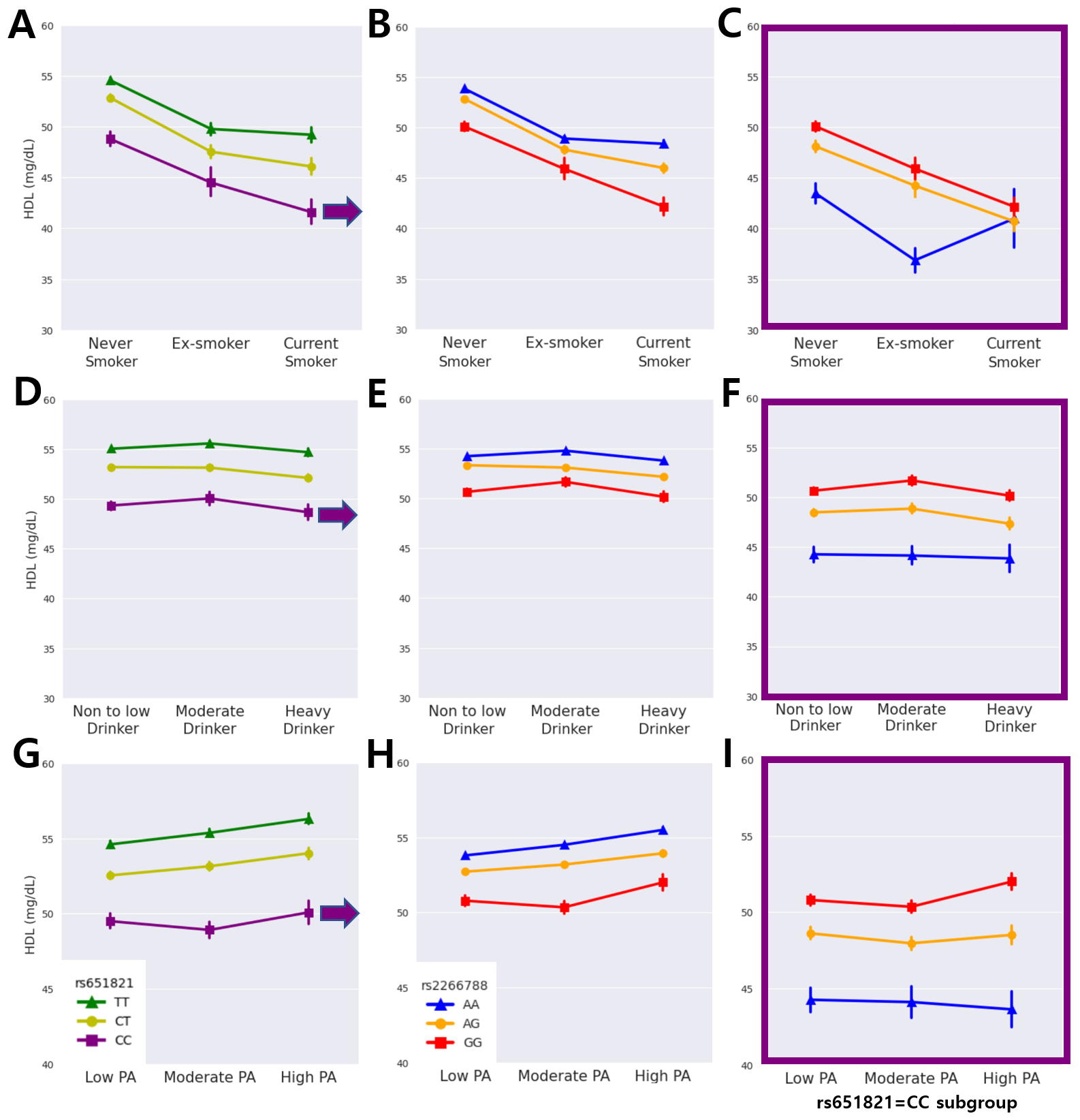


Plasma HDL levels in accordance to rs651821 and rs2266788 genotypes stratified by smoking **(A-C)**, alcohol drinking **(D-F)**, and physical activity **(G-I)**. Subfigures **A, D, G** correlates to rs651821 genotypes in the total population, **B, E, H** correlates to rs2266788 genotypes in the total population and **C, F, I** refers to rs2266788 genotypes within the rs651821 CC subgroup. HDL: High-density lipoprotein cholesterol, PA: Physical activity
